# Hypertonic saline alleviates cerebral edema by inhibiting microglia-derived TNF-α and IL-1β-induced Na-K-Cl Cotransporter up-regulation

**DOI:** 10.1186/1742-2094-11-102

**Published:** 2014-06-11

**Authors:** Lin-Qiang Huang, Gao-Feng Zhu, Yi-Yu Deng, Wen-Qiang Jiang, Ming Fang, Chun-Bo Chen, Wei Cao, Miao-Yun Wen, Yong-Li Han, Hong-Ke Zeng

**Affiliations:** 1 Southern Medical University, Guangzhou 510515, PR China; 2 Department of Emergency & Critical Care Medicine, Guangdong General Hospital, Guangdong Academy of Medical Sciences, Guangzhou 510080, PR China

## Abstract

**Background:**

Hypertonic saline (HS) has been successfully used clinically for treatment of various forms of cerebral edema. Up-regulated expression of Na-K-Cl Cotransporter 1 (NKCC1) and inflammatory mediators such as tumor necrosis factor alpha (TNF-α) and interleukin-1 beta (IL-1β) has been demonstrated to be closely associated with the pathogenesis of cerebral edema resulting from a variety of brain injuries. This study aimed to explore if alleviation of cerebral edema by 10% HS might be effected through down-regulation of inflammatory mediator expression in the microglia, and thus result in decreased NKCC1 expression in astrocytes in the cerebral cortex bordering the ischemic core.

**Methods:**

The Sprague-Dawley (SD) rats that underwent right-sided middle cerebral artery occlusion (MCAO) were used for assessment of NKCC1, TNF-α and IL-1β expression using Western blotting, double immunofluorescence and real time RT-PCR, and the model also was used for evaluation of brain water content (BWC) and infarct size. SB203580 and SP600125, specific inhibitors of the p38 and JNK signaling pathways, were used to treat primary microglia cultures to determine whether the two signaling pathways were required for the inhibition of HS on microglia expressing and secreting TNF-α and IL-1β using Western blotting, double immunofluorescence and enzyme-linked immunosorbent assay (ELISA). The effect of TNF-α and IL-1β on NKCC1 expression in primary astrocyte cultures was determined. In addition, the direct inhibitory effect of HS on NKCC1 expression in primary astrocytes was also investigated by Western blotting, double immunofluorescence and real time RT-PCR.

**Results:**

BWC and infarct size decreased significantly after 10% HS treatment. TNF-α and IL-1β immunoexpression in microglia was noticeably decreased. Concomitantly, NKCC1 expression in astrocytes was down-regulated. TNF-α and IL-1β released from the primary microglia subjected to hypoxic exposure and treatment with 100 mM HS were decreased. NKCC1 expression in primary astrocytes was concurrently and progressively down-regulated with decreasing concentration of exogenous TNF-α and IL-1β. Additionally, 100 mM HS directly inhibited NKCC1 up-regulation in astrocytes under hypoxic condition.

**Conclusions:**

The results suggest that 10% HS alleviates cerebral edema through inhibition of the NKCC1 Cotransporter, which is mediated by attenuation of TNF-α and IL-1β stimulation on NKCC1.

## Background

Cerebral edema results from various cerebral insults, such as ischemic stroke [1] and traumatic brain injury [2,3]. Hypertonic saline (HS) has been widely used for the treatment of patients with traumatic shock, cerebral edema and elevated intracranial pressure (ICP) resulting from cerebral infarction, hemorrhage or traumatic brain injury [4,5]. The various types of edema result from permeability changes induced by multiple factors affecting the brain’s cellular barriers [6]. It is well known that HS removes free water from the intracellular into the extracellular space through osmotic force and reduction of peripheral vascular resistance [7]. Our previous study has shown that in addition to its osmotic force, 10% HS exerts anti-edema effects possibly through down-regulation of AQP4 expression in the cerebral cortex astrocytes in the ischemic cerebral edema [8]. This suggests that ion channel transporters related to water transport whose expression is localized in astrocytes and other cerebral cell types are potential therapeutic targets in HS treatment.

The Na-K-Cl cotransport systems, which consist of two isoforms (NKCC1 and NKCC2), have been shown to play an important role in ion homeostasis and the subsequent accumulation of intracellular water [9,10]. The transcriptional up-regulation of Na-K-Cl Cotransporter 1 (NKCC1) in the blood-brain barrier, choroid plexus and neuroglial cells contributes to these permeability changes [6,11]. Ischemia-triggered cytotoxic edema is due to the entry of sodium into neuroglial cells via electroneutral ion transporters like NKCC1 [12]. It has been found that sodium, chloride and other solutes influx intracellularly caused by up-regulated NKCC1 results in cell swelling [12-14]. This is why NKCC1 plays an important role in astrocyte swelling/cerebral edema in ischemia and trauma [15,16]. Some studies have shown that administration of the NKCC1 blocker bumetanide can attenuate the cell swelling and injury, suggesting that sodium and chloride transport via NKCC1 is involved in ischemia-induced cell swelling and injury [17,18]. A milder gray and white matter damage has been found in NKCC1 knockout mice after focal cerebral ischemia [19]. Therefore, inhibition of NKCC1 expression could alleviate cerebral edema and protect neurologic functions effectively.

In addition, a previous study has shown that NKCC1 could be selectively up-regulated by TNF-α and IL-1β [20]. It was suggested that the relationship between NKCC1 and pro-inflammatory cytokines, such as TNF-α and IL-1β, may be one of the key factors of cerebral edema.

Microglia are the innate immune cells residing in the central nervous system (CNS), and they serve as the brain’s immune defense. They are readily activated in different stress stimuli, such as inflammation and hypoxia. Previous studies have shown that cytokines, such as TNF-α and IL-1β released from microglia under hypoxic-ischemic and inflammation conditions [21,22], are closely related to cerebral edema because they can disrupt the endothelial’s tight junction [22,23]. Inhibition of microglia activation is beneficial to cerebral edema. It has been reported that HS treatment is beneficial as it attenuates inflammation by suppressing neutrophil activation [24-26] via inhibiting the P38 MAPK pathway.

Arising from the above, we hypothesized that 10% HS administration could decrease the production of TNF-α and IL-1β released by microglia under ischemia-hypoxic condition. As a corollary, down-regulating the NKCC1 expression in the cerebral cortex astrocytes in the peri-ischemic brain tissue would ameliorate the cerebral edema. If this were proven to be so, it would be pertinent to extending the study to investigate the potential underlying mechanism whereby HS could affect microglial release of TNF-α and IL-1β.

## Methods

### Animals and experimental groups

A total of 156 male Sprague-Dawley (SD) rats weighing 220 to 250 g were randomly divided into a sham-operated group (abbreviated: sham group, *n* = 52), cerebral ischemic + normal saline group (abbreviated: ischemic group, *n* = 52) and cerebral ischemic + 10% HS treatment group (abbreviated: 10% HS group, *n* = 52). The whole operation process followed the aseptic surgical techniques, the tail vein was cannulated to facilitate the intravenous (i.v.) infusion of 10% HS or normal saline. Rats in the ischemic group and 10% HS group were subjected to right-sided middle cerebral artery occlusion (MCAO), sham-operated rats were subjected to the surgical procedures except for MCAO.

After operation, rats in the sham group were treated with a continuous i.v. infusion of normal saline (0.3 ml/h) via the tail vein until the end of the experiment. At 2 h following MCAO, rats in the ischemic group and 10% HS group were treated with a continuous i.v. infusion of normal saline (0.3 ml/h) and 10% HS, respectively. On the basis of different treatment times, animals were further subdivided into two groups: 12 h and 24 h subgroups. The number of rats killed at various time points in different groups is shown in Table 1. Animal handling and experiments were approved by Institutional Animal Care and Use Committee, Guangdong Province, China.

**Table 1:** Number of rats killed at various time points in different groups

	**BWC measurement**	**TTC staining**	**Double immunofluorescence**	**Real time RT-PCR**	**Western blotting**
**Sham-operated group**					
**12 h**	7	0	10	6	6
**24 h**	7	6	10	0	0
**Cerebral ischemic group**					
**12 h**	7	0	10	6	6
**24 h**	7	6	10	0	0
**Cerebral ischemic + 10% HS group**					
**12 h**	7	0	10	6	6
**24 h**	7	6	10	0	0

BWC, brain water content; TTC, 2,3,5-triphenyltetrazolium chloride.

### Focal brain ischemia animal model and neurological deficit evaluation

Before surgical procedure, the rats were fasted overnight, but were allowed free access to water. The rats were anesthetized with 5% ketamine (40 mg/kg) (FuJian GuTian Pharmaceutical Co., Ltd., Ningde, FuJian, China). A heating lamp was used to maintain the rectal temperature between 37 and 37.5°C throughout the surgical procedures. Focal brain ischemia was induced using the intraluminal suture MCAO method as described in detail in our previous study [8]. The rats in the sham-operated group were subjected to the surgical procedures except for intravascular filament occlusion. At 2 h after MCAO, the intraluminal filament was carefully pulled out to establish reperfusion and the neurologic deficit was evaluated. The neurologic findings were scored on a five-point scale based on the Zea-Longa scale [27]: 0, no observable neurologic deficit; 1, a mild focal neurologic deficit (failing to extend left forepaw fully); 2, a moderate focal neurologic deficit (circling to the left); and 3, a severe focal deficit (falling to the left); 4, the rats did not move or walk spontaneously and had a depressed level of consciousness. The rats with a neurologic deficit score of 1 to 3 were considered effective models.

### TTC assessment of infarct size

A total of 18 male SD rats were randomly divided into a sham-operated group (n = 6), a cerebral ischemic + normal saline group (n = 6) and a cerebral ischemic + 10% HS treatment group (n = 6). At 24 h after MCAO, the rats were killed with 5% ketamine anesthesia, the brains were rapidly removed and frozen at -70°C for three minutes. After this, 3 mm coronal slices were prepared with a rat brain matrix (68709; RWD Life Technology Co., Ltd, Shenzhen, China) starting at the frontal pole. Slices were incubated with 2% 2,3,5,-triphenyltetrazolium chloride monohydrate (TTC; Sigma, St. Louis, MO, USA) for 30 minutes at 37°C protected from light and flipped every five minutes for consistent staining of anterior and posterior faces [15,28]. The sections were then scanned. The cerebral infarct area in each section was calculated by Image-Pro Plus 6.0 (Media Cybernetics, Inc. Rockville, MD, USA). Infarct areas on each section were summed and multiplied by section thickness to give the infarction volume.

### Assessment of ischemic hemispheric cerebral edema

Under deep anesthesia condition, rats were killed at the end of the experiment by decapitation (n = 7 per group). The brains were quickly removed and blotted gently to remove small quantities of adsorbent moisture. After this, the brains were dissected into ipsilateral ischemic and contralateral hemispheres through the interhemispheric fissure. The ratio of wet to dry weight was used to estimate cerebral edema [29]. Tissues were immediately weighed on an electronic balance with a scale to within 0.001 mg. Dry weight was measured after the ischemic hemispheres were heated for three days at 100°C in a drying oven. Tissue water content was then calculated as% H2O = (1 - dry weight/wet weight) × 100% [29].

### Mixed glia cell culture

Mixed glia cultures were prepared from a modified method based on the method of Giulian and Baker [30]. In brief, cerebral hemispheres were separated from 0 to 24 h-old SD pups, the meninges and superficial vessels were carefully removed. The cerebral cortex was harvested and digested with 10 ml 0.125% trypsin containing 600 U DNase (Sigma, St. Louis, MO, USA, Cat. No. D4527) for 15 minutes in 37°C thermostatic water bath. After this, 10 ml of Dulbecco’s modified Eagle’s medium-F12 nutrient mixture (DMEM-F12) (Invitrogen Life Technologies Corporation, Carlsbad, CA, USA; Cat. No. 31330-038) containing 10% fetal bovine serum (FBS) (Invitrogen Life Technologies Corporation; Cat. No. 10099-141) was added. The tissue was then triturated several times with a 5 ml pipette. The un-dissociated tissue clumps were allowed to settle for one to two minutes. Subsequently, the supernatant was collected and passed through a 70 μm cell strainer to remove the remaining small clumps of tissue; the cell suspension was then centrifuged at 1,500 rpm for five minutes. The supernatant was discarded and the cell pellet was resuspended in 10 ml of DMEM-F12 supplemented with 10% FBS. The resuspended cells were seeded into poly-L-lysine-coated 75 cm^2^ flasks at a density of 250,000 cells/ml and cultured at 37°C in humidified 5% CO_2_/95% air. The medium was changed after 24 h and then replaced every three to four days.

### Microglia purification and treatment

After 10 to 14 days, the bottom of the flask showed a confluency of cells with mixed glia, including mainly astrocytes/microglia/oligodendrocytes. A high purity microglia was harvested by the shaking method of Giulian and Baker [30]. The flasks were placed in an orbital shaker at 37°C and shaken at 180 rpm for two hours. The supernatant was collected and centrifuged at 1,500 rpm for five minutes. For enzyme-linked immunosorbent assay (ELISA), cells were plated on poly-L-lysine-coated 24-well plates at a density of 2.5 × 10^5^ cells per well; for Western blotting, cells were plated on poly-L-lysine-coated six-well plates at a density of 1.5 × 10^6^ cells per well. After incubation in a humidified atmosphere of 95% air and 5% CO_2_ at 37°C for 24 h, the cells were subjected to different treatments. About 96% of cells in cultures were identified to be microglia by staining with Iba-1 antibody (Table 2), a specific marker of microglia.

**Table 2:** Antibodies used in experiments

**Antibody**	**Host**	**Company**	**Cat. No.**	**Application**	**Concentration**
**GFAP**	Mouse	Millipore, Billerica, MA, USA	MAB360	Tissue/cell (IHC, IF, WB)	1:100
**GFAP**	Mouse	Santa Cruz Biotechnology, Santa Cruz, CA, USA	sc-58766	Cell (IF)	1:200
**OX42**	Mouse	Millipore, Billerica, MA, USA	CBL1512Z	Tissue (IHC)	1:50
**Iba-1**	Mouse	abcam, Cambridge, MA, USA	ab15690	Cell (IF)	1:200
**NKCC1**	Rabbit	Millipore, Billerica, MA, USA	AB 3560P	Tissue/cell (IHC, IF, WB)	1:100
**NKCC1**	Goat	Santa Cruz Biotechnology, Santa Cruz, CA, USA	sc-21545	Cell (IF, WB)	1:50
**TNF-α**	Rabbit	Millipore, Billerica, MA, USA	AB1837P	Tissue (IHC, WB)	1:50
**TNF-α**	Rabbit	Chemicon International, Temecula, CA, USA	AB1837P	Cell (IF)	1:400
**IL-1β**	Rabbit	Abcam, Cambridge, MA, USA	ab9787	Tissue (IHC, WB)	1:100
**IL-1β**	Rabbit	Chemicon International, Temecula, CA, USA	AB1832P	Cell (IF)	1:400
**Phos-p38**	Rabbit	Cell Signaling Technology, Danvers, MA, USA	4511S	Cell (WB)	1:1,000
**P38**	Rabbit	Cell Signaling Technology, Danvers, MA, USA	8690S	Cell (WB)	1:1,000
**Phos-JNK**	Rabbit	Cell Signaling Technology, Danvers, MA, USA	4668S	Cell (WB)	1:1,000
**JNK**	Rabbit	Cell Signaling Technology, Danvers, MA, USA	9252S	Cell (WB)	1:1,000
**β-actin**	Rabbit	Cell Signaling Technology, Danvers, MA, USA	4970	Cell (WB)	1:1,000
**GAPDH**	Goat	Santa Cruz Biotechnology, Santa Cruz, CA, USA	sc-20357	Tissue (WB)	1:1,000

IF, immunofluorescent; IHC, Immunohistochemistry; WB, Western blotting.

To study whether HS would affect microglial release of inflammatory mediators as well as its underlying mechanism, microglial cells were treated with (HS group) or without (Hypoxia group) 100 mM HS for 4 h at 3% oxygen, 5% CO2 and 92% nitrogen at 37°C. To explore whether HS would affect the P38 and JNK signaling pathways in microglia, another two groups of cells were added: SB203580 group and SP600125 group. SB203580 (10 μM, Sigma, Cat. No. S8307) and SP600125 (10 μM, Sigma, Cat. No. S5567), specific inhibitors of the P38 and JNK signaling pathways, respectively, were used to compare the effect of HS on microglial release of TNF-α and IL-1β. After different treatments, the cultured medium was collected and stored at -80°C for ELISA.

To study the effect of HS on the P38 and JNK signaling pathways under hypoxic condition, microglia cells were divided into three groups: control group, HS group, Hypoxia group. The HS group and Hypoxia group were further subdivided into four groups according to different treatment times: 30 minutes, 1, 2 and 4 h subgroups. Subsequently, the protein was extracted from the microglial cells and stored at -80°C for Western blotting. In all the experiments, cells were cultured in glucose-free medium except for the control which was cultured in DMEM-F12 containing 10% FBS.

### Astrocyte purification and treatment

After 10 to 14 days, astrocytes were purified by shaking [31]. Astrocytes were isolated by trypsinization. For Western blotting and mRNA extraction, cells were seeded in a six-well plate at a density of 5 × 10^5^ cells per well and incubated at 95% air and 5% CO_2_ for 24 h. Following this, the medium was replaced by glucose free medium + TNF-α (PeproTech, Rocky Hill, NJ, USA, Cat. No. 400–14) (5 ng/ml or 10 ng/ml) or IL-1β (PeproTech, Cat. No. 400-01B) (5 ng/ml or 10 ng/ml) and cultured at 37°C in humidified 5% CO_2_ and 95% air for 4 h, and the cells were extracted for protein or mRNA.

TNF-R and interleukin-1 receptor (IL-1R) antagonists (TNF-α antagonist III, R-7050, Santa Cruz Biotechnology, Santa Cruz, CA, USA; Cat. No. sc-356159 and IL-1R antagonist, Santa Cruz Biotechnology; Cat. No. sc-221747), respectively, were used to ascertain the role of TNF-α and IL-1β on NKCC1 expression in astrocytes. Cells were pre-incubated for 30 minutes with TNF-α antagonist (2 μM) and IL-1R antagonist (2 μM) followed by TNF-α (10 ng/ml) and IL-1β (10 ng/ml) treatment.

To ascertain whether HS had a direct effect on NKCC1 mRNA and protein in astrocytes under hypoxic condition, astrocytes were cultured in glucose-free medium and treated with or without HS (100 mM) at 3% oxygen, and 5% CO_2_ at 37°C. Bumetanide (0.1 mM, B3023, Sigma), a specific inhibitor of NKCC1, was used as a negative control. The three groups were named the hypoxia group, HS group and bumetanide group, respectively.

### Western blotting

**Figure 1:**
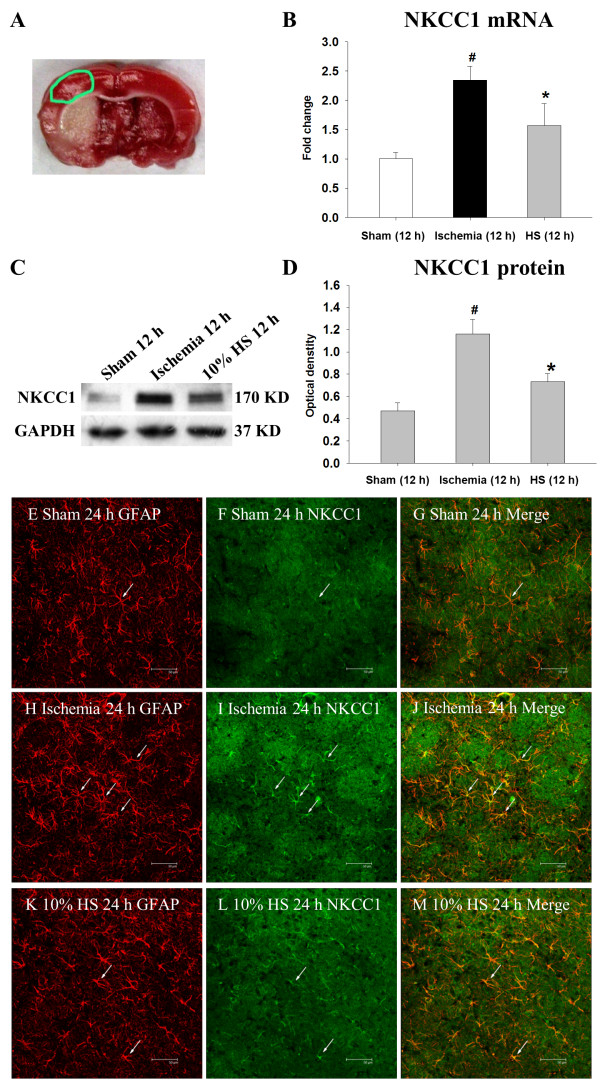
**Ten percent HS down-regulated NKCC1 expression in peri-ischemic brain tissue.** Sample was extracted from the peri-ischemic brain tissue outlined by the green line in **(A)**. NKCC1 mRNA and protein expression in the peri-ischemic brain tissue of ischemic and ischemic + 10% HS rats at 12 h following MCAO and the corresponding control rats (sham-operated). Section **(B)** shows the graphical representation of the fold changes in NKCC1 mRNA in each group as quantified by normalization to the β-actin as an internal control (n = 6 per group). Section **(C)** shows NKCC1 (170 kDa) and GAPDH (37 kDa) immunoreactive bands, respectively. **(D)** This is a bar graph showing significant changes in the relative optical density of NKCC1 to GAPDH in each group. Significant differences in mRNA and protein levels in the peri-ischemic brain tissue of ischemic 12 h, ischemic 12 h + 10% HS are evident when compared with sham-operated ( # *P* <0.05,* *P* <0.05*,* n = 6 per group). Confocal images showing the distribution of GFAP labeled (**E, H, K,** red), and NKCC1 (**F, I, L**, green) immunoreactive astrocytes (arrows) in the peri-ischemic brain tissue of ischemic and ischemic + 10% HS rats at 24 h following MCAO and the corresponding control rats (sham-operated). GFAP labeling clearly overlaps NKCC1 immunofluorescence in (**G, J** and **M**). Note that NKCC1 expression in the perivascular astrocytes (arrows) is markedly enhanced following MCAO. After treatment with 10% HS, NKCC1 expression is noticeably attenuated. Scale bars: (E-M), 50 μm. GFAP, glial fibrillary acidic protein; HS, hypertonic saline; NKCC1, Na-K-Cl Cotransporter 1; MCAO, middle cerebral artery occlusion.

Proteins were extracted from the peri-ischemic brain tissue in each group (n = 6) (Figure 1A), microglia cells and astrocytes using a total protein extraction kit (Bei Jing Pu Li Lai Gene Technology Co., Ltd., China) according to the manufacturer’s protocol. Protein concentrations were determined by the BCA method [32] using BCA-100 protein quantitative analyzing kit (Shanghai Biocolor Bioscience & Technology Co. Ltd., Shanghai, China), and then the protein samples were heated at 95°C for five minutes and separated by SDS-PAGE. The proteins were blotted onto polyvinylidene difluoride membrane and then blocked with 5% non-fat milk for 1 h at room temperature. An overnight incubation with the primary antibodies followed the manufacturer’s recommendations. After rinses with Tris-buffered saline with 0.1% Tween-20, the membranes were incubated with the horseradish peroxidase (HRP) -conjugated secondary antibodies for 1 h at room temperature. The primary antibodies for the tissue used were as follows: TNF-α, IL-1β and NKCC1. The primary antibodies for the cells used (Table 2) were as follows: P38, Phos-P38, JNK, Phos-JNK and NKCC1. The secondary antibodies used were as follows: goat anti-rabbit IgG-HRP (1:3,500, Cell Signaling Technology, Cat. No.7074), donkey anti-goat IgG-HRP (1:5,000, Santa Cruz Biotechnology; Cat. No. sc-2020). The immunoblots were developed using the enhanced chemiluminescence detection system (Bei Jing Pu Li Lai Gene Technology Co., Ltd., China). The signal intensity was measured with FluorChem 8900 software (version 4.0.1, Alpha Innotech Corporation, San Leandro, CA, USA), and fold change was calculated.

### Real time RT-PCR

Total RNA was extracted from the peri-ischemic brain tissue (n = 6 per group) (indicated in Figure 1. A with green line) for detection of NKCC1, TNF-α and IL-1β mRNA at 12 h after MCAO using Trizol Reagent (Invitrogen Life Technologies Corporation, USA) according to the manufacturer’s protocol. The quantity of total RNA was measured with a UV Spectrophotometer (Biochrom Ltd., UK). For the reverse transcription, 2 μl of total RNA was combined with 4 μl of 5 × PrimeScript® Buffer (PrimeScript® RT Master Mix, TaKaRa Biotechnology (Dalian) Co., Ltd., China; Cat No: DRR036S). RNase Free dH2O was added to 20 μl, after which the mixture was heated at 37°C for 15 minutes, 85°C for 5 s. Quantitative RT-PCR was carried out on a Light Cycler 480 instrument using a FastStart DNA Master plus SYBR Green I kit (Roche Diagnostics, GmbH, Roche Applied Science, Penzberg, Germany) according to the manufacturer’s instructions. The expression of target genes was measured in triplicate, and was normalized to β-actin as an internal control. The oligonucleotide primer sequences used were as follows: NKCC1 (160 bp), Forward: TGATTCCACTTCCTTTATTGCAG, Reverse: TTAATGAG TTGAGCTCCGGTGA; TNF-α (134 bp), Forward: CCAACAAGGAGGAGAAGTTCC, Reverse: CTCTGCTTGGTGGTTTGCTAC; IL-1β (123 bp), Forward: GGAACCCGT GTCTTCCTAAAG, Reverse: CTGACTTGGCAGAGGACAAAG; β-actin (203 bp), Forward: GCCAACACAGTGCTGTCTG, Reverse: TACTCCTGCTTGCTGATCCA. Gene expression was quantified using a modification of the 2^-ΔΔCT^ method as previously described [33].

For astrocytes, the fold change of NKCC1 was also measured after treatment with different concentrations of TNF-α and IL-1β for 4 h. The procedure was carried out as mentioned above.

### Analysis of TNF-α and IL-1β expression by ELISA

After microglia cells were subjected to hypoxia for 4 h, the cultured medium from each group was collected. The levels of TNF-α and IL-1β released by microglia were then measured using TNF-α (IBL, Hamburg, Germany, Cat. No. JP27194) and IL-1β (IBL, Minneapolis, MN, USA, Cat. No. 27193) ELISA kit according to the manufacturer’s protocol. Finally, the reaction plates were read within 30 minutes in an enzyme micro-plate reader at 450 nm.

### Double immunofluorescence

Double immunofluorescence staining was carried out to detect TNF-α and IL-1β expression in microglia, and NKCC1 expression in astrocytes in peri-ischemic brain tissue. Five rats in each group were deeply anesthetized with an overdose of 5% ketamine and perfused transcardially with 4% paraformaldehyde in 0.1 M phosphate-buffered saline (PBS) (pH 7.4). Coronal frozen sections of the brain of 5 μm thickness at the level of the optic chiasma were cut and rinsed in PBS (n = 5 per group). Endogenous peroxidase activity was blocked with 0.3% hydrogen peroxide in methanol for 20 minutes followed by rinsing with PBS. After this, sections were incubated with a blocking solution (5% normal goat serum, 0.3% Triton X-100 in PBS) for 30 minutes at 37°C. The tissue slices were then incubated overnight with NKCC1 and glial fibrillary acidic protein (GFAP), or OX42 and TNF-α/IL-1β. On the following day, the sections protected from light were washed and incubated with secondary antibodies: Alexa Fluor® 488 goat anti-rabbit IgG (H + L) (1:200; Invitrogen Life Technologies Corporation; Cat. No. A-11008) and Alexa Fluor® 594 goat anti-mouse IgG (1:200; Invitrogen Life Technologies Corporation; Cat. No. A-11005) for 1 h at room temperature. Following rinsing in PBS, they were mounted with a fluorescent mounting medium (DAKO Cytomation, Glostrup, Denmark). Cellular co-localization was observed in a confocal microscope (Leica TCS SP2 AOBS; Leica Microsystems Ltd., Wetzlar, Germany).

After hypoxia for 4 h, purified microglia were fixed in 4% paraformaldehyde in 0.1 M PBS (pH 7.4) for 20 minutes, blocked with 5% normal goat serum for 30 minutes, and then incubated with a mixture of Iba-1 and TNF-α or IL-1β overnight. Subsequent antibody detection was carried out with secondary antibodies: goat anti-mouse IgG-FITC (1:200, Santa Cruz Biotechnology, Cat. No. sc-2010) and Goat Anti-Rabbit IgG (H + L)-cy3 (1:200, earthOX, San Francisco, CA, USA) for 1 h. Finally, the cells were examined under a fluorescence microscope (Olympus DP73 Microscope, Olympus, Tokyo, Japan).

Purified astrocytes were treated with TNF-α or IL-1β. The subsequent procedure was carried out as mentioned above. The primary antibodies included GFAP and NKCC1, the secondly antibodies were R-PE-conjugated AffiniPure F(ab’)2 Fragment Donkey An7-Mouse IgG (H + L) (1:200, Chicago, IL, USA, Cat. No. SA00008-9) and chicken anti-goat IgG-FITC (1:200, Santa Cruz Biotechnology, Cat. No. sc-2988), or Alexa Fluor® 488 Goat Anti-Mouse IgG (H + L) (1:200; Invitrogen Life Technologies Corporation; Cat. No. A-11001) Antibody and Alexa Fluor® 555 Goat Anti-Rabbit IgG (H + L) (1:200; Invitrogen Life Technologies Corporation; Cat. No. A-21428). Finally, the cells were examined under a fluorescence microscope (Olympus DP73 Microscope, Olympus, Tokyo, Japan).

### Statistical analysis

All data were evaluated by the SPSS13.0 statistical software (IBM, Armonk, New York, USA). Different statistical methods were applied according to different types of data. The distribution values were expressed as mean ± SD. Three-group two-factor measurement data were analyzed by two-way classification ANOVA. Three-group univariate-factor measurement data were analyzed by one-way ANOVA if the data were homogeneity of variance; otherwise, they were analyzed by Welch ANOVA. Multiple comparisons were analyzed by the least significant difference (LSD) method if the data were homogeneity of variance; otherwise they were analyzed by Dunnett’s T3 method. The criterion for statistical significance was set at *P* <0.05.

## Results

### Neurologic deficit score

**Figure 2:**
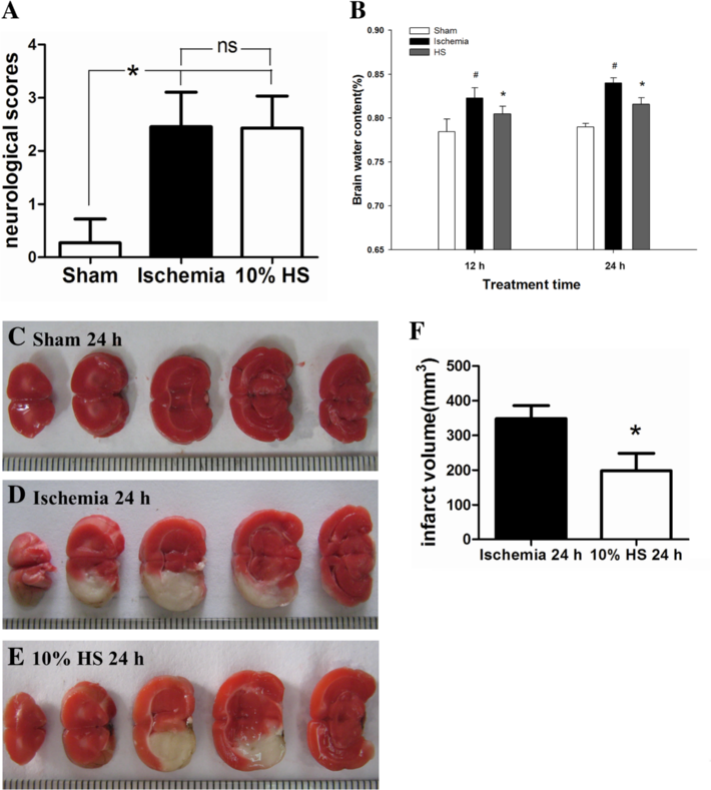
**The effect of 10% HS on BWC and infarct size.** Bar graph **A** shows that the neurological score shows no difference between the ischemia group and the 10% HS group (ns: *P* >0.05*,* n = 7 per group). Bar graph **B** shows that the percentage of BWC is significantly increased in the ipsilateral ischemic hemisphere at 12 h and 24 h following MCAO when compared with corresponding controls (sham-operated) (# *P* <0.05). However, it is significantly decreased in the ipsilateral ischemic hemisphere in 10% HS groups as compared with corresponding ischemic group (* *P* <0.05*,* n = 7 per group). TTC staining images **(C-E)** shown are from three representative experiments. The white area represents the infarct brain, and the red area represents the normal brain. Bar graph **F** shows the infarct volume is significantly decreased in 10% HS group when compared with ischemia group (* *P* <0.05, n = 6 per group). Note 10% HS could reduce infarct size effectively. Scale bars: (C-E), 1 mm per compartment. BWC, brain water content; HS, hypertonic saline; MCAO, middle cerebral artery occlusion; TTC, 2, 3, 5-triphenyltetrazolium chloride.

Compared with the sham-operated group, the Zea-Longa scores in the ischemic group and the 10% HS group at the beginning of reperfusion increased significantly as analyzed by the LSD method (Figure 2A) (*P <0.05). However, there was no difference in the Zea-Longa scores before intervention between the ischemic group and the 10% HS group (Figure 2A) (*P* = 0.997).

### Ipsilateral ischemic hemispheric brain water content (BWC) and infarct size

BWC in the ipsilateral ischemic hemispheres was significantly different among the sham group, the ischemic group and the 10% HS group (*F* = 56.620, *P* <0.05). There was no interaction between treatment time and treatment factors (*F* = 1.079, *P* = 0.351). Differences in treatment factors among different groups did not change over time. The separate effects of treatment factors were further analyzed. When compared with that in the sham group, BWC in the ischemic cerebral hemispheres increased significantly in the ischemic group and 10% HS group at 12 h and 24 h analyzed by the LSD method (Figure 2B) (#*P* <0.05). Compared with the ischemic group, BWC in the ischemic cerebral hemisphere decreased significantly in the 10% HS group at 12 h and 24 h. This showed that treatment of ischemic rats with 10% HS alleviated the ipsilateral ischemia-induced increase in BWC at 12 and 24 h after MCAO (Figure 2B) (*P <0.05).

TTC staining images showed that the infarct size in the 10% HS group (Figure 2E) was smaller than that in the ischemic group (Figure 2D). Cerebral infarct volume was significantly decreased in the 10% HS group in comparison to the cerebral ischemic group (Figure 2F) (*P <0.05). The data demonstrated that 10% HS administration exerts a neuroprotective effect on cerebral ischemia.

### NKCC1 mRNA and protein expression in the peri-ischemic brain tissue

NKCC1 mRNA expression was significantly increased in the peri-ischemic brain tissue at 12 h following MCAO rats in comparison with the sham-operated rats (Figure 1B) (#*P* <0.05). However, after treatment with 10% HS, NKCC1 mRNA expression in the peri-ischemic brain tissue was significantly decreased when compared with the matching ischemic rats (Figure 1B) (*P <0.05). The immune-reactive bands of NKCC1 protein levels that appeared at approximately 170 kDa increased significantly in optical density at 12 h following MCAO were compared with the sham-operated group (Figure 1C-D) (#*P* <0.05). However, after treatment with 10% HS, the optical density was decreased significantly when compared with the matching ischemic rats (Figure 1C,D) (*P <0.05).Intense NKCC1 immunoreactivity was detected in the astrocytes in the peri-ischemic brain tissue at 24 h following MCAO (Figure 1H-J) when compared with the controls (Figure 1E-G). On the other hand, after treatment with 10% HS, NKCC1 immunoreactivity in the astrocytes in the peri-ischemic brain tissue (Figure 1K-M) was markedly decreased when compared with the matching ischemic rats.

### TNF-α and IL-1β mRNA and protein expression in peri-ischemic brain tissue

**Figure 3:**
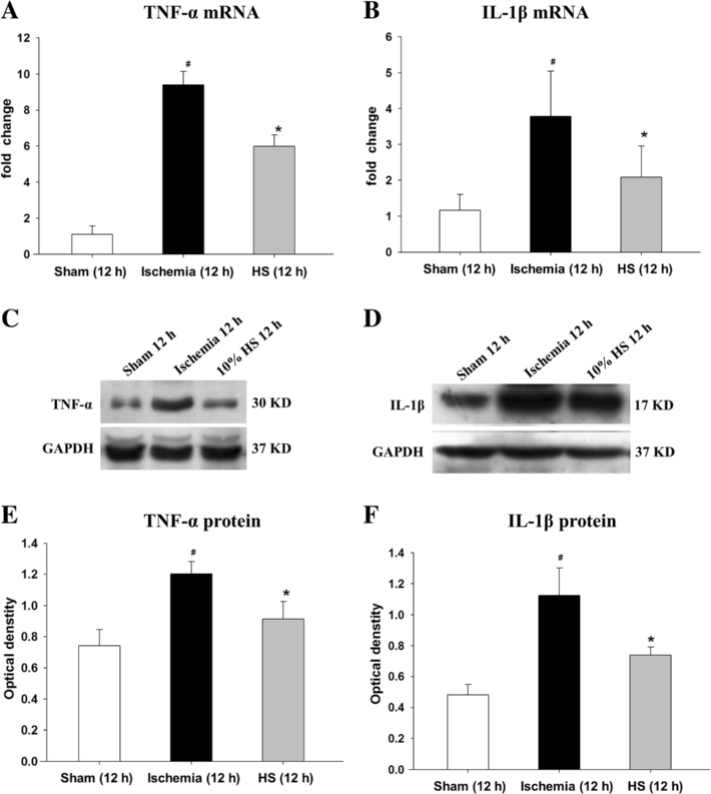
**TNF-α and IL-1β mRNA and protein expression in the peri-ischemic brain tissue.** Panels **A** and **B** show the graphical representation of the fold changes in TNF-α and IL-1β mRNA, respectively. They show that 10% HS significantly decreased TNF-α and IL-1β mRNA expression at 12 h after MCAO. Panels **C** and **D** show TNF-α (30 kDa), IL-1β (17 kDa) and GAPDH (37 kDa) immunoreactive bands, respectively. Panels **E** and **F** show the optical density of TNF-α and IL-1β expression in an ischemic 12-h group is significantly up-regulated when compared with the sham-operated group (#*P* <0.05*,* n = 6 per group); however, the optical density of TNF-α and IL-1β in the ischemic 12-h + 10% HS group is significantly decreased after 10% HS treatment (*P <0.05*,* n = 6 per group). IL-1β, interleukin-1 beta; HS, hypertonic saline; MCAO, middle cerebral artery occlusion; TNF-α, tumor necrosis factor-alpha.

Both TNF-α and IL-1β mRNA expression was significantly increased (Figure 3A-B) (#*P* <0.05) in ischemic group as compared with sham group at 12 h, but declined after treatment with 10% HS in the 10% HS group (Figure 3A,B) (*P <0.05). TNF-α and IL-1β protein expression was increased significantly (Figure 3C-F) (#*P* <0.05) at 12 h following MCAO in comparison with the sham group, but after treatment with 10% HS, both TNF-α and IL-1β expression were markedly decreased as compared with ischemic group (Figure 3C-F) (*P <0.05).

### Cellular localization and expression of TNF-α and IL-1β in the peri-ischemic brain tissue

**Figure 4:**
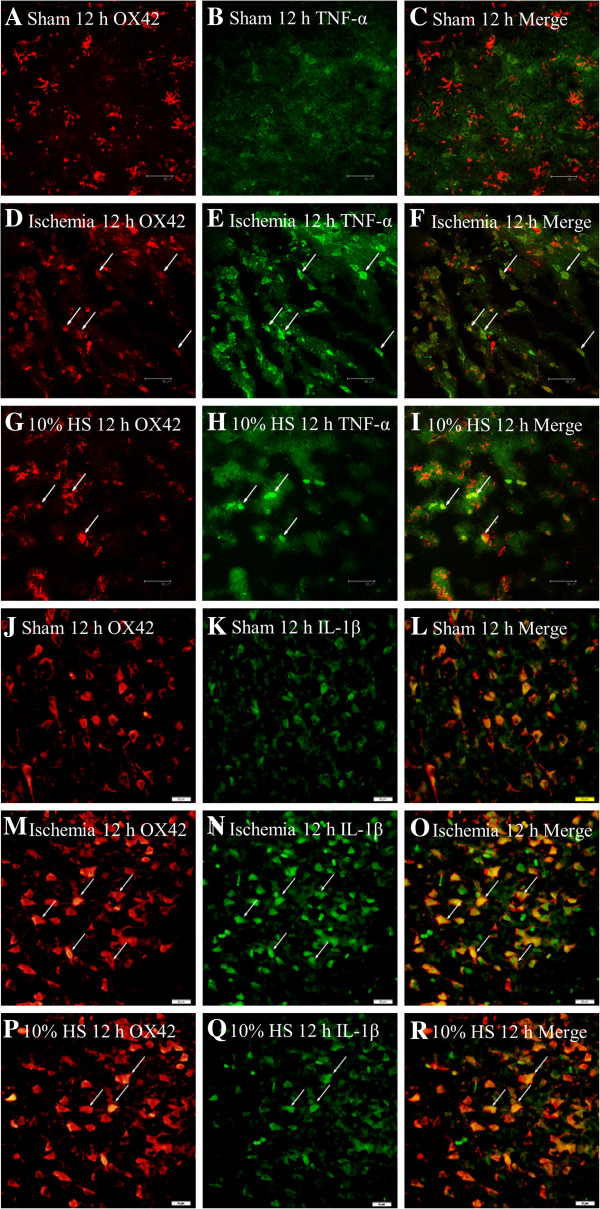
**Ten percent HS reduced the TNF-α and IL-1β expression in microglia-located peri-ischemic brain tissue.** Confocal images showing the distribution of OX42 labeled microglia (**A, D, G, J, M, P**, red), TNF-α (**B, E, H**, green) and IL-1β (**K, N, Q**, green) in peri-ischemic brain tissue at 12 h after MCAO and the corresponding control rats. OX42 labeling overlapping TNF-α immunofluorescence can be seen in **C, F** and **I**, OX42 labeling overlaps IL-1β immunofluorescence in **L, O** and **R**. Note that TNF-α and IL-1β expression in microglia cells (arrows) is markedly enhanced following MCAO. However, after treatment with 10% HS, they are noticeably reduced. Scale bars: **(A–R)**, 50 μm. HS, hypertonic saline; IL-1β, interleukin- 1beta; MCAO, middle cerebral artery occlusion; TNF-α, tumor necrosis factor-alpha.

In the sham group (Figure 4A-C, Figure 4J-L), only moderate TNF-α and IL-1β expression was detected in some small cells with multiple branched processes and confirmed to be the microglia by double immunofluorescence with OX42. Confocal images showed that TNF-α and IL-1β immunoreactivity was significantly increased (Figure 4D-F, Figure 4M-O) in ischemic group at 12 h after MCAO when compared with sham group (Figure 4A-C, Figure 4J-L). However, TNF-α and IL-1β expression was markedly reduced in HS group following treatment of 10% HS (Figure 4G-I, Figure 4P-R).

### TNF-α and IL-1β protein expression in primary activated microglia under hypoxic condition

**Figure 5:**
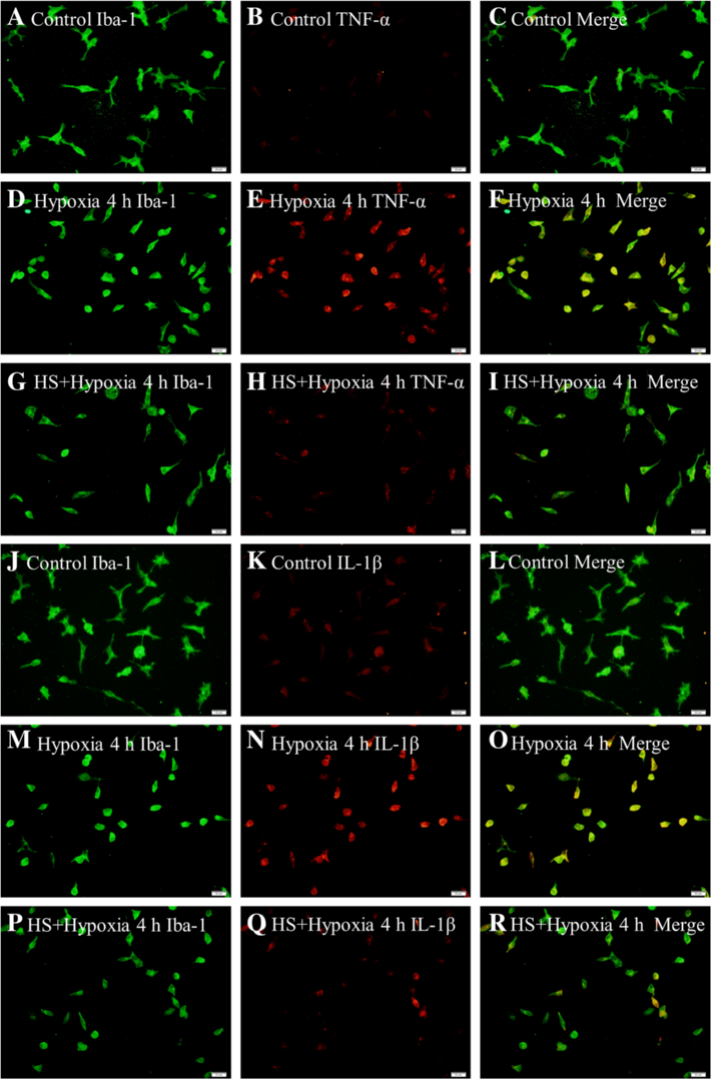
**HS attenuated hypoxia-induced TNF-α and IL-1β expression in microglia.** Immunofluorescence images of cultured microglia showing the expression of Iba-1 (**A, D, G, J, M, P**, green), TNF-α (**B, E, H**, red) and IL-1β (**K, N, Q**, red). The co-localized expression of Iba-1 and TNF-α can be seen in panels D, **F, I**. The co-localized expression of Iba-1 and IL-1β can be seen in panels **L, O, R**. Note that TNF-α and IL-1β expression are markedly down-regulated in microglia after treatment with 100 mM HS as compared with the hypoxia group cells. Scale bars: (A-R), 20 μm. HS, hypertonic saline; IL-1β, interleukin- 1beta; TNF-α, tumor necrosis factor-alpha.

At 4 h after hypoxia, TNF-α and IL-1β immunoexpression in primary microglia in hypoxia group was markedly enhanced (Figure 5D-F, Figure 5M-O) compared with that in the control group (Figure 5A-C, Figure 5J-L). On the other hand, in the HS group (Figure 5G-I, Figure 5P-R), TNF-α and IL-1β immunoexpression was evidently decreased. In addition, when compared with that in the hypoxia group, more rounded microglia were observed in the hypoxia group (Figure 5D-F, Figure 5M-O) than that in the HS group (Figure 5G-I, Figure 5P-R).

**Figure 6:**
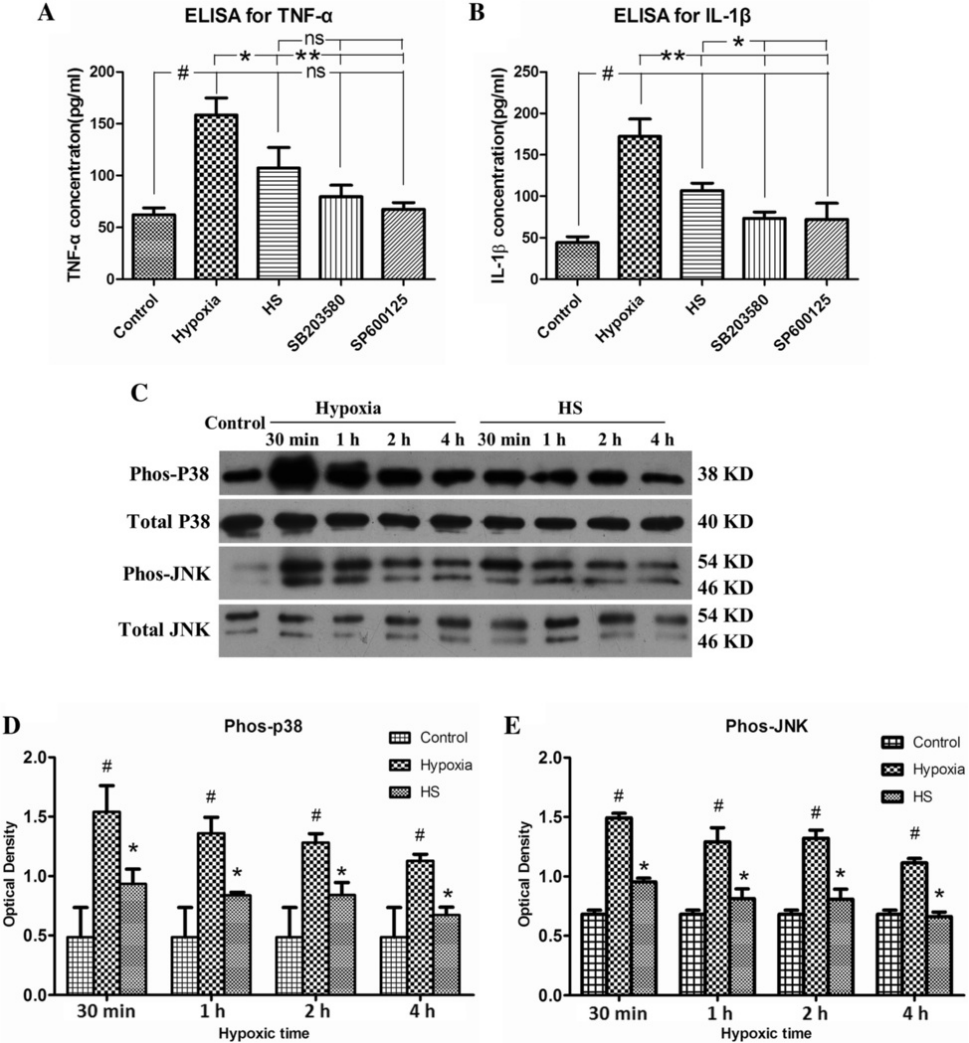
**HS attenuated hypoxia-induced TNF-α and IL-1β expression by inhibiting the activation of p38 and JNK signaling pathways.** Panels **A** and **B** show levels of TNF-α **(A)** and IL-1β **(B)** protein in cultured medium from microglia decreased significantly after treatment with 100 mM HS, SB203580 and SP600125, as compared with the hypoxia group (*P < 0.05, **P < 0.01 ). Panel **C** shows p38, JNK phosphorylation and total p38, JNK immunoreactive bands. Bar graphs **D** and **E** show the optical density of phosphorylated p38 (38 kDa) and JNK (46 kDa, 54 kDa) immunoreactive bands are significantly increased at 30 minutes, 1 h, 2 h and 4 h following hypoxia, respectively (#*P* < 0.05). However, after treatment with 100 mM HS, the optical density of phosphorylated p38 and JNK is significantly decreased as compared with the corresponding hypoxia groups (*P < 0.05). ns: non-significant, *P* > 0.05. HS, hypertonic saline; IL-1β, interleukin- 1beta; TNF-α, tumor necrosis factor-alpha.

The levels of TNF-α and IL-1β in cultured medium were also measured by ELISA. The results showed that TNF-α and IL-1β release from microglia was significantly elevated (Figure 6A,B) (#*P* <0.05) after hypoxia as compared with that in the control group. However, the levels of TNF-α and IL-1β were significantly decreased (Figure 6A,B) (*P <0.05, ** *P* <0.01) after treatment with 100 mM HS.

### The role of MAP kinase signaling pathway in microglial release of TNF-α and IL-1β

ELISA showed a significant increase in TNF-α and IL-1β levels at 4 h after hypoxia when compared with the control group (Figure 6A,B) (#*P* <0.05), but the levels were markedly decreased after SB203580 (a specific P38 MAPK inhibitor) or SP600125 (a specific JNK inhibitor) treatment as compared with the hypoxia group (Figure 6A,B) (*P <0.05, **P <0.01). The same effect was observed after 100 mM HS treatment, indicating that P38 and JNK may be involved in inflammatory mediators released by microglia. Therefore Western blot was performed to examine whether HS could directly affect the MAPK and JNK signaling pathway in microglia. The results showed that both phos-MAPK and phos-JNK protein expression levels were significantly down-regulated following HS treatment at 30 minutes, 1 h, 2 h and 4 h under hypoxic conditions when compared with the hypoxia group (Figure 6C-E) (*P <0.05).

### NKCC1 mRNA and protein expression in primary astrocytes after treatment with TNF-α, IL-1β and TNF-R and IL-1R antagonists

**Figure 7:**
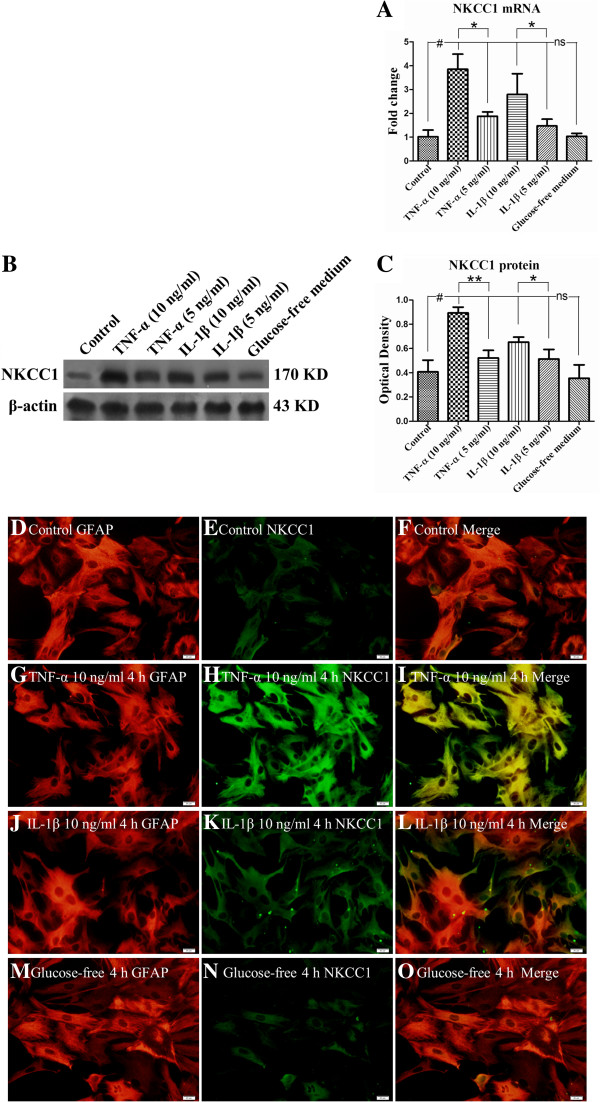
**TNF-α or IL-1β dose-dependently up-regulated NKCC1 mRNA and protein expression in primary astrocytes.** Bar graph **A** shows both TNF-α and IL-1β significantly elevated (#*P* < 0.05) NKCC1 mRNA expression. Panels **B** and **C** depicting TNF-α (10 ng/ml or 5 ng/ml) or IL-1β (10 ng/ml or 5 ng/ml) treatment protein significantly increased NKCC1 protein when compared with that in the control group (#*P* <0.05). The alteration of NKCC1 expression is dose dependent, that is, a higher concentration of TNF-α or IL-1β induced more NKCC1 mRNA and protein expression (A-C) (*P < 0.05, **P < 0.01). Immunofluorescence images of astrocytes showing GFAP labeled astrocytes (**D, G, J, M**, red), co-labeled with NKCC1 (**E, H, K, N**, green). The co-localized expression of GFAP and NKCC1 can be seen in panels **F, I, L** and **O**. Note NKCC1 expression is significantly up-regulated in astrocytes after treatment with a higher concentration of TNF-α (10 ng/ml) or IL-1β (10 ng/ml) as compared with the control group. ns: non-significant, *P* > 0.05. Scale bars: **(D–O)**, 20 μm. GFAP, glial fibrillary acidic protein; IL-1β, interleukin-1beta; NKCC1, Na-K-Cl Cotransporter 1; TNF-α, tumor necrosis factor-alpha.

**Figure 8:**
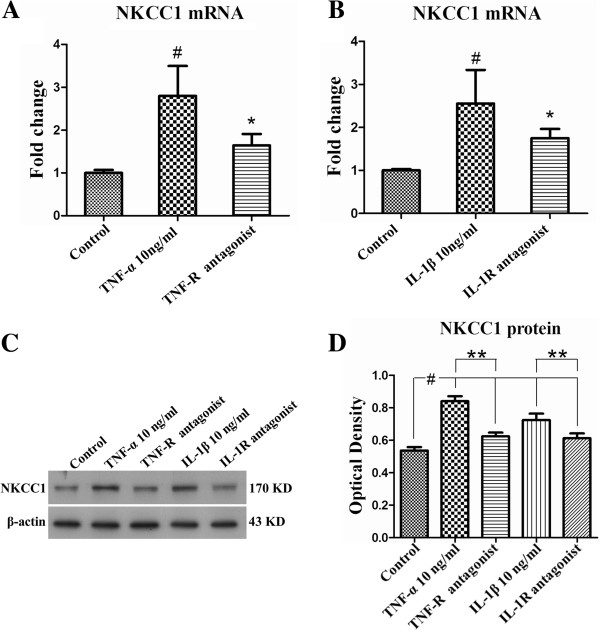
**The effect of TNF-R and IL-1R antagonists on NKCC1 mRNA and protein expression in astrocytes.** Bar graphs **A** and **B** show that NKCC1 mRNA expression in astrocytes is significantly down-regulated by TNF-R and IL-1R antagonists (*P < 0.05). Panel **C** shows NKCC1 (170 kDa) and β-actin (43 kDa) immunoreactive bands, respectively. Bar graph **D** shows significant reduction in the optical density of NKCC1 protein following TNF-R and IL-1R antagonist treatment (**P < 0.01). IL-1R, interleukin-1 receptor; NKCC1, Na-K-Cl Cotransporter 1; TNF-α, tumor necrosis factor-alpha.

**Figure 9:**
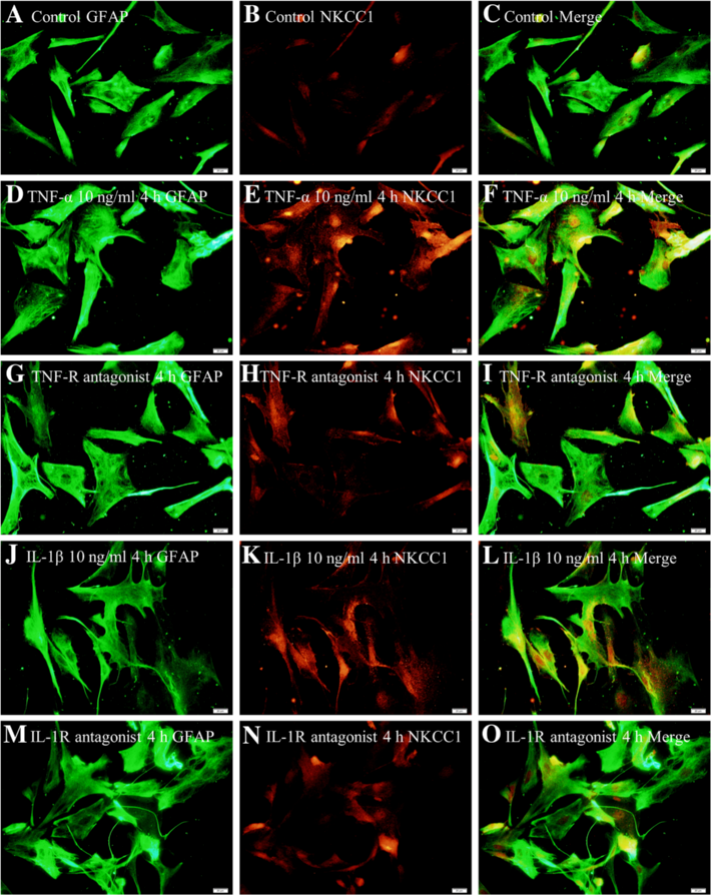
**NKCC1 expression in astrocytes after TNF-R and IL-1R antagonist treatment.** Immunofluorescence images show the expression of GFAP (**A, D, G, J, M**, green), and NKCC1 (**B, E, H, K, N**, red). Co-localized expression of GFAP and NKCC1 can be seen in **C, F, I, L** and **O**. Note that TNF-R and IL-1R antagonist could revert the effect of TNF-α and IL-1β on NKCC1 expression. Scale bars: (A–O), 20 μm. GFAP, glial fibrillary acidic protein; IL-1R, interleukin-1 receptor; NKCC1, Na-K-Cl Cotransporter 1; TNF-α, tumor necrosis factor-alpha.

To ascertain whether the TNF-α and IL-1β played a role in NKCC1 mRNA and protein expression, astrocytes were treated with glucose-free medium containing different doses of TNF-α (10 ng/ml or 5 ng/ml) or IL-1β (10 ng/ml or 5 ng/ml), TNF-R (2 μM) and IL-1R antagonists (2 μM). It was found that both TNF-α and IL-1β could significantly elevate (Figure 7A-C) (#*P* <0.05) the expression of NKCC1 mRNA and protein when compared with the control group and glucose-free medium group. This effect appeared to be dose-dependent; thus, the higher the concentration of TNF-α or IL-1β, the greater the expression of NKCC1 mRNA and protein (Figure 7A-C) (*P <0.05, **P <0.01). More strikingly, NKCC1 was drastically decreased in astrocytes when the cells were pre-incubated with TNF-R and IL-1R antagonist (Figure 8A-D) (*P <0.05, ** *P* <0.01).Double immunofluorescence showed that more intense NKCC1 immunoreactivity was detected in primary astrocytes after treatment with TNF-α (10 ng/ml) (Figure 7G-I) and IL-1β (10 ng/ml) (Figure 7J-L), when compared with the control group (Figure 7D-F) and the glucose-free medium group (Figure 7M-O). However, both TNF-R and IL-1R antagonists could significantly revert the NKCC1 immunoreactivity in astrocytes (Figure 9G-I, M-O) treated with TNF-α (Figure 9D-F) or IL-1β (Figure 9J-L).

### NKCC1 mRNA and protein in astrocytes under hypoxic conditions

**Figure 10:**
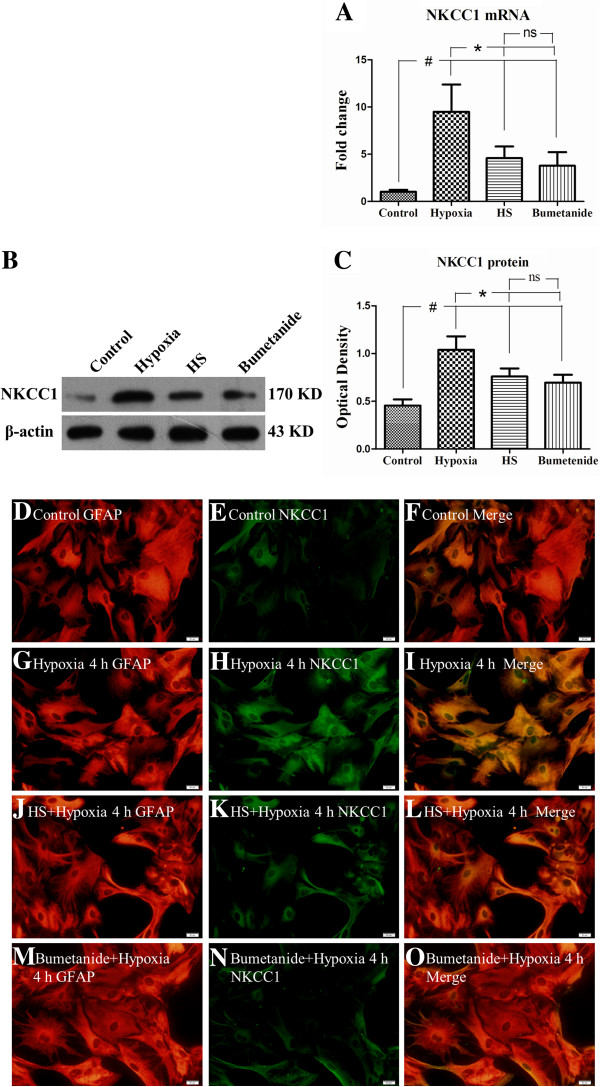
**HS may directly down-regulate NKCC1 mRNA and protein expression in primary astrocytes under hypoxic condition.** Bar graph **A** shows that NKCC1 mRNA expression in primary astrocytes decreased significantly (*P < 0.05) following treatment with HS or bumetanide under hypoxic conditions. Panels **B** and **C** show the optical density of NKCC1 protein is significantly increased following hypoxic exposure as compared with control group (#*P* < 0.05); however, NKCC1 protein expression is markedly reduced (*P < 0.05) after treatment with HS or bumetanide (a specific NKCC1 inhibitor). Immunofluorescence images of astrocytes showing the expression of GFAP-labeled astrocytes (**D, G, J, M**, red), co-labeled with NKCC1 (**E, H, K, N**, green). The co-localized expression of GFAP and NKCC1 can be seen in panels **F, I , L** and **O**. Note NKCC1 expression is down-regulated in astrocytes after treatment with 100 mM HS **(K)** as compared with hypoxia group cells (H). ns: non-significant, *P* > 0.05. Scale bars: (D-L), 20 μm. GFAP, glial fibrillary acidic protein; HS, hypertonic saline; NKCC1, Na-K-Cl Cotransporter 1.

The results showed that at 4 h after hypoxia, NKCC1 mRNA (Figure 10A) and protein (Figure 10B,C) expression was significantly increased in hypoxia group as compared with the control group (#*P* <0.05). However, following treatment with 100 mM HS or 0.1 mM bumetanide, NKCC1 mRNA (Figure 10A) and protein (Figure 10B,C) expression was drastically decreased in comparison with the hypoxia group (*P <0.05).In parallel with mRNA and protein expression, the immunoreactivity of NKCC1 was significantly increased in primary astrocytes following hypoxic exposure (Figure 10G-I), whereas this was weak and moderate in the control group (Figure 10D-F). However, either 100 mM HS or 0.1 mM bumetanide pretreatment, NKCC1 immunoreactivity of HS group (Figure 10J-L) and bumetanide group (Figure 10M-O) was noticeably attenuated as compared with the hypoxia group.

## Discussion

Osmotherapy agents, such as hypertonic saline and mannitol, are currently being used for treatment of cerebral edema and elevated ICP resulting from ischemic stroke [1]. Hypertonic saline has recently received considerable attention as an alternative to mannitol because it is more effective in ameliorating cerebral edema than an equal volume of 20% mannitol [34]. The present study has shown that ipsilateral ischemic hemispheric BWC increased significantly following right-sided MCAO for 12 h, suggesting an acute and early onset of cerebral edema after ischemic insults. After treatment with 10% HS for 12 and 24 h, BWC of the ipsilateral ischemic hemisphere decreased significantly. This suggests that 10% HS can alleviate cerebral edema effectively. Our results are consistent with previous studies [7].

Inappropriate activation and increased expression of NKCC1 will contribute to cell swelling and tissue edema. Endothelial NKCC1 was also activated after ischemia and this process was associated with astrocyte swelling/cerebral edema [28,35]. The astrocyte swelling and cerebral edema were effectively reduced by bumetanide, a specific inhibitor of NKCC1 [36,37]. Our results have shown an up-regulation of the mRNA and protein expression of NKCC1 in the peri-ischemic brain tissue. Double immunofluorescence staining has further demonstrated that NKCC1 expression was localized in the cerebral cortex astrocytes as verified by its co-localization with GFAP, a specific cellular marker for astrocytes. More importantly, it has also been found that 10% HS treatment alleviated the NKCC1 expression in the cerebral cortex astrocytes with the reduction of BWC. In light of the above, it is suggested that besides its osmotic force, osmotherapy with 10% HS can attenuate cerebral edema probably through down-regulation of NKCC1 expression in the cerebral cortex astrocytes. Remarkably, the infarct size in the peri-ischemic brain tissue was drastically reduced following 10% HS treatment. These results indicated that 10% HS administration can alleviate ischemic brain damage with the reduction of cerebral edema and, hence, it is neuroprotective.

Microglia is extremely sensitive to hypoxic-ischemia [38], and it is well documented that microglial activation is a hallmark feature in different cerebral diseases. Under ischemic-hypoxic conditions, a variety of inflammatory cytokines, such as TNF-α and IL-1β [21], are released by activated microglia. These inflammatory cytokines have been reported to interrupt the blood brain barrier (BBB) by destroying the endothelial tight junctions, thus leading to cerebral edema [23]. In addition, reactive alterations in morphology, changing from ramified into “ameboid” phenotype [39], was observed and this is indicative of microglial activation. Previous studies have shown that reduced TNF-α and IL-1β caused a marked reduction in ischemic brain damage and cerebral edema [40,41]. This suggests that inhibition or suppression of microglial activation and release of TNF-α and IL-1β might ameliorate BBB disruption and cerebral edema. The present results demonstrated that TNF-α and IL-1β mRNA and protein levels were significantly increased at 12 h following MCAO. By RT-PCR, Western blot analysis and double immunofluorescence study, it was demonstrated that 10% HS could reduce the levels of TNF-α and IL-1β expression by microglia. *In vitro* results were consistent with *in vivo* experiments. Thus, at 4 h after hypoxia, not only the microglial morphology was altered from resting into an activated state, but the release of TNF-α and IL-1β in the culture medium from the primary microglia was also increased. However, the production of TNF-α and IL-1β was markedly reduced after treatment with 100 mM HS. These results suggest that HS can exert an inhibitory effect on microglia in its release of TNF-α and IL-1β. The question arising from this would be what is the underlying mechanism regulating the inhibitory effect on inflammation of HS.

It is well documented that the P38 and JNK signaling pathways are closely related to cytokine production. We therefore used SB203580 and SP600125, specific inhibitors of JNK and p38, respectively, to explore whether P38 and JNK signaling pathways would be involved in TNF-α and IL-1β production in primary microglia. The present results have demonstrated that both SB203580 and SP600125 could cause a marked reduction of TNF-α and IL-1β release by microglia, supporting that P38 and JNK are involved in the production of TNF-α and IL-1β. Because the inhibitory effect is in concert with that of HS, and hypertonicity may have an effect of inhibition on P38 which have been shown in previous studies [25,42], we next investigated whether HS had an effect on phosphorylated P38 and JNK expression in microglia. Remarkably, phosphorylated P38 and JNK levels were significantly up-regulated after hypoxia for 4 h, and were significantly down-regulated after HS treatment. Taken together, it is concluded that activated microglia release high levels of TNF-α and IL-1β via P38 and JNK signaling pathways under hypoxic condition, but most strikingly HS could interrupt this process by inhibiting phosphorylation of P38 and JNK proteins.

It has been reported by others that hypertonicity could activate P38 and JNK signaling pathways in neutrophils [26,43], but it was also pointed out that HS could not activate P38 at the Na^+^ at concentration < 200 mM. For the JNK signaling pathway, the concentration of HS that activated the JNK signaling pathway was more than 400 mM [24]. In this study, the concentration of HS used was 100 mM *in vitro.* Even if we were to consider the additional Na^+^ in DMEM, the concentration of HS would be approximately 200 mM. Therefore, HS inhibition of the P38 and JNK signaling pathways as demonstrated in the present results may be through intercepting signaling pathways upstream [24,25,42]. The other consideration is that a low concentration of HS can only weakly activate P38 but it can prevent a strong activation by other stimuli, such as hypoxia. Further studies are necessary to clarify the mechanism underlying this phenomenon.

The next issue to be resolved would be the relationship among TNF-α and IL-1β and NKCC1. It has been reported that NKCC1 expression in microvascular endothelial cells from rats and human umbilical vein endothelial cells is selectively up-regulated by TNF-α and IL-1β [20]. *In vitro*, the present results show that NKCC1 mRNA and protein expression in primary astrocytes was significantly augmented when incubated with TNF-α or IL-1β, but was down-regulated with declining concentration of TNF-α or IL-1β. Furthermore, TNF-R and IL-1R antagonist could revert the increased expression of NKCC1 induced by TNF-α and IL-1β. These results suggest that TNF-α and IL-1β may directly up-regulate NKCC1 expression. Therefore, NKCC1 down-regulation after MCAO following HS treatment may partially be attributed to decreased TNF-α and IL-1β production.

We also investigated whether HS had a direct inhibitory effect on NKCC1 expression in primary astrocytes. The results have shown that NKCC1 mRNA and protein expression levels were lower in the HS group as compared with the hypoxia group, suggesting that HS could directly inhibit NKCC1 expression in astrocytes. This result appears to contradict the finding that hyperosmotic could activate NKCC1 [44]. A possible explanation for this discrepancy would be a differential response to HS between different cell types. The other possible explanation may be that HS decreases viability of astrocytes and protein content because of its excessive osmotic pressure (200 mOsm (100 mM HS) + 310 mOsm (glucose-free medium)). In view of this, the blood osmotic pressure and Na^+^ concentration in clinical application of HS should be considered.

Taken together, 10% HS treatment ameliorates ischemic cerebral edema through reducing the production of TNF-α and IL-1β released by microglia. This would result in reduced NKCC1 expression in astrocytes. It is also noteworthy that HS may also directly down-regulate NKCC1 expression in astrocytes.

## Conclusion

We show here that 10% HS ameliorated ischemic cerebral edema and infarct size in the peri-ischemic brain tissue. Concomitantly, 10% HS down-regulated increased NKCC1 expression in the cerebral cortex astrocytes as well as TNF-α and IL-1β released by activated microglia. The relation between NKCC1 expression and TNF-α and IL-1β was unequivocally demonstrated *in vitro* using TNF-R and IL-1R antagonists. Furthermore, HS decreased TNF-α and IL-1β released by microglia by inhibiting the activation of P38 and JNK signaling pathway, thus reducing the stimulation on NKCC1. These results demonstrated that in addition to its osmotic force, 10% HS exerts anti-edema effects possibly through down-regulation of NKCC1 expression in the cerebral cortex astrocytes in the ischemic cerebral edema. A total of 10% HS administration may be neuroprotective in view of its exertive effect in reducing the cerebral edema. This study leaves us with a new concept as to the mechanism of 10% HS treatment, that is, aside from setting up an osmotic gradient, HS ameliorates ischemic cerebral edema not merely by down-regulating aquaporin AQP4 expression but also that of NKCC1. This would provide a better theoretical basis for clinical use of HS.

## Abbreviations

AQP4: Aquaporin-4; BWC: Brain water content; CCA: Common carotid artery; CSF: Cerebrospinal fluid; DMEM-F12: Dulbecco’s modified eagle medium/nutrient mixture F-12; ECA: External carotid artery; FBS: Fetal bovine serum; GFAP: Glial fibrillary acidic protein; HRP: Horseradish peroxidase; HS: Hypertonic saline; ICA: Internal carotid artery; ICP: Intracranial pressure; i.v.: intravenous; IL-1β: Interleukin-1 beta; MCA: Middle cerebral artery; MCAO: Middle cerebral artery occlusion; NKCC1: Na-K-Cl cotransporter 1; PBS: Phosphate buffered saline; SD: Sprague-Dawley; TBI: Traumatic brain injury; TJ: Tight junction; TNF-α: Tumor necrosis factor alpha; TTC: 2,3,5-triphenyltetrazolium chloride.

## Competing interests

The authors declare that they have no competing interests.

## Authors’ contributions

ZGF carried out assessment of the NKCC1 expression in the cerebral cortex astrocytes by Western blotting, Real-time RT-PCR, collected data and drafted the manuscript. HLQ participated in study of the mechanism of HS’s effect on microglia releasing TNF-α and IL-1β and drafted the manuscript. DYY participated in the design of the study and drafted the manuscript. JWQ carried out assessment of infarct size by TTC staining in the ischemic hemisphere and ischemic hemispheric cerebral edema. FM participated in assessment of the NKCC1 expression in the cerebral cortex astrocytes by double immunofluorescence. CCB performed the statistical analysis. CW participated in making the focal brain ischemia animal model. WMY participated in assessment of the effect of TNF-α and IL-1β on NKCC1 expression in primary astrocytes. HYL carried out assessment of the effect of HS on NKCC1 expression in primary astrocytes. ZHK carried out the design of the study and performed the statistical analysis. All authors read and approved the final manuscript.
